# Non-structural carbohydrate dynamics and growth in tomato plants grown at fluctuating light and temperature

**DOI:** 10.3389/fpls.2022.968881

**Published:** 2022-10-03

**Authors:** Ana Cristina Zepeda, Ep Heuvelink, Leo F. M. Marcelis

**Affiliations:** Horticulture and Product Physiology, Department of Plant Sciences, Wageningen University, Wageningen, Netherlands

**Keywords:** non-structural carbohydrates, soluble sugars, starch, plant growth, temperature fluctuations, light fluctuations, carbon storage, structural growth

## Abstract

Fluctuations in light intensity and temperature lead to periods of asynchrony between carbon (C) supply by photosynthesis and C demand by the plant organs. Storage and remobilization of non-structural carbohydrates (NSC) are important processes that allow plants to buffer these fluctuations. We aimed to test the hypothesis that C storage and remobilization can buffer the effects of temperature and light fluctuations on growth of tomato plants. Tomato plants were grown at temperature amplitudes of 3 or 10°C (deviation around the mean of 22°C) combined with integration periods (IP) of 2 or 10 days. Temperature and light were applied in Phase (high temperature simultaneously with high light intensity, (400 μmol m^–2^ s^–1^), low temperature simultaneously with low light intensity (200 μmol m^–2^ s^–1^) or in Antiphase (high temperature with low light intensity, low temperature with high light intensity). A control treatment with constant temperature (22°C) and a constant light intensity (300 μmol m^–2^ s^–1^) was also applied. After 20 days all treatments had received the same temperature and light integral. Differences in final structural dry weight were relatively small, while NSC concentrations were highly dynamic and followed changes of light and temperature (a positive correlation with decreasing temperature and increasing light intensity). High temperature and low light intensity lead to depletion of the NSC pool, but NSC level never dropped below 8% of the plant weight and this fraction was not mobilizable. Our results suggest that growing plants under fluctuating conditions do not necessarily have detrimental effects on plant growth and may improve biomass production in plants. These findings highlight the importance in the NSC pool dynamics to buffer fluctuations of light and temperature on plant structural growth.

## Introduction

In a natural environment, plants are exposed to strong fluctuations in light intensity and temperature which lead to periods of asynchrony between carbon (C) supply by photosynthesis and C demand by the plant organs (Palacio et al., 2014). The storage and remobilization of non-structural carbohydrates (NSC) are important processes that allow plants to buffer these fluctuations. Plants distribute recent carbon assimilated by the leaves (sources) into various carbon sinks such as growth, metabolic maintenance, storage and defense (Chapin et al., 1990). In many plant species, NSC are accumulated as soluble sugars and starch (Martínez-Vilalta et al., 2016) when the net production of carbohydrates exceeds the demand and growth is limited by assimilate usage (Palacio et al., 2014). The remobilization of stored C compounds is fundamental for plants to support growth and metabolism during periods of limited C supply (Dietze et al., 2014; Chuste et al., 2020), for example at higher latitudes in winter when short light periods and low light intensity are typical (Bours, 2014) or in perennial plants when there are no leaves that can do photosynthesis. Remobilization can occur across time scales: from diurnal (day-night) (Smith and Stitt, 2007; Graf et al., 2010), over day-to-day (influenced by the weather on that specific day), up to seasonal remobilization (Furze et al., 2018). Despite the central role of storage and remobilization of C in response to fluctuations in light and temperature, plant responses to repeated changes in the carbon supply and demand, generated, for example, by repeatedly changing the light intensity or growth temperature are largely unknown.

On a time scale of weeks, plants can adjust rates of respiration (Atkin and Tjoelker, 2003) or photosynthesis (Yamori et al., 2014) to compensate for changes in temperature or light environment through an adjustment in physiology, structure or biochemistry of the leaves (Smith and Dukes, 2013). These acclimation responses have been extensively studied, usually by growing plants at specific temperatures or light conditions and then exposing those plants to a new growth condition for several days. Under excess light and low temperatures, accumulation of NSC (mainly as starch and soluble sugars) is typical as an acclimation response in the long term (days) (Ruelland et al., 2009; Thalmann and Santelia, 2017; Pommerrenig et al., 2018). Lower temperatures also have an immediate effect on enzymatic activity which leads to a reduction in photosynthetic capacity (Huner et al., 1993; Ruelland et al., 2009) and respiration (Atkin and Tjoelker, 2003). Higher light levels may result in an increased accumulation of carbohydrates in the leaves, causing decreased expression of photosynthetic genes that lead to a downregulation in photosynthesis (Paul and Foyer, 2001). At low light intensities, the net production of assimilates is less than the net demand of assimilates; making that plant growth is limited by C supply (Smith and Stitt, 2007). If low light intensities coincide with higher temperatures, this may lead to a depletion of the NSC pool because of the increased energy requirements for respiration and growth (conversion into structural C) (Atkin and Tjoelker, 2003). So, while the effects of prolonged high supply or low demand of C on plant growth and development are well established, we know little of how much of the accumulated NSC pool is available for remobilization once the plants face more favorable environmental conditions and at what time scales accumulation and remobilization do occur (Dietze et al., 2014; Furze et al., 2018; Wiley et al., 2019). A change from warm to cool conditions and vice versa showed that tomato plants grown at low temperatures increased the NSC concentration over a week and then almost completely remobilized within 12 h exposure to warm temperatures (Klopotek and Kläring, 2014). Besides, the effect of dynamic light and temperature fluctuations at a scale of 1 to 2 days on the dynamic response of processes such as growth and storage is still largely unexplored.

Although C has a central role in plants, our understanding of its daily dynamics of storage and remobilization under short term (days) and long term (weeks) climate fluctuations is still limited. A quantitative understanding of C storage and remobilization is essential in order to explain plant responses to temporal suboptimal climate conditions. The overall aim of this paper was to evaluate how plants accumulate and remobilize C in response to short term (every other day) and long-term (every 10 days) temperature and light fluctuations and how these fluctuations affect growth and morphology. Plants are capable of storing carbohydrates under conditions where growth is demand-limited, and later on remobilize this carbon when temperature rises up to a certain limit. Consequently, we hypothesize that this C storage and remobilization buffers the effects of temperature and light fluctuations on growth of tomato plants.

## Materials and methods

### Plant materials and growth conditions

Tomato (*Solanum lycopersicum* “Moneymaker”) seeds were sown on stonewool plugs in a climate cabinet at 22°C, relative humidity 70% and 200 μmol m^–2^ s^–1^ 16 h photoperiod. After 15 days, seedlings were transplanted to (7 × 7 cm) stonewool cubes (Rockwool Grodan, Roermond, Netherlands) and distributed over three climate cabinets with the same climate conditions as mentioned above. The growth surface of each climate cabinet was 0.84 m^2^. Light was provided by white LED modules (GreenPower LED-TL-DR/W-MBVISN 0.16/0.24/0.59 blue/green/red fraction, Philips, Eindhoven, Netherlands). The height of the LED lamps was adjusted weekly to maintain the desired photosynthetic photon flux density (PPFD) at the top of the canopy. Climate in the cabinet was controlled by a climate control computer. Plants were watered with nutrient solution (electrical conductivity 2.1 dS m^–1^, pH 5.5) containing 1.2 mM NH_4_^+^, 7.2 mM K^+^, 4.0 mM Ca^2+^, 1.8 mM Mg^2+^, 12.4 mM NO_3_^–^, 3.3 mM SO_4_^2–^, 1.0 mM PO_4_^2–^, 35 μM Fe^3+^, 8.0 μM Mn^2+^, 5.0 μM Zn^2+^, 20 μM B, 0.5 μM Cu^2+^, 0.5 μM MoO_4_^2–^. After 28 days after sowing (DAS) six plants were taken from each cabinet and destructively measured. Remaining plants were re-distributed over four cabinets. Treatments started in all four climate cabinets with a plant density of 59 plants m^–2^ and lasted 20 days.

### Treatments and experimental set up

**FIGURE 1:**
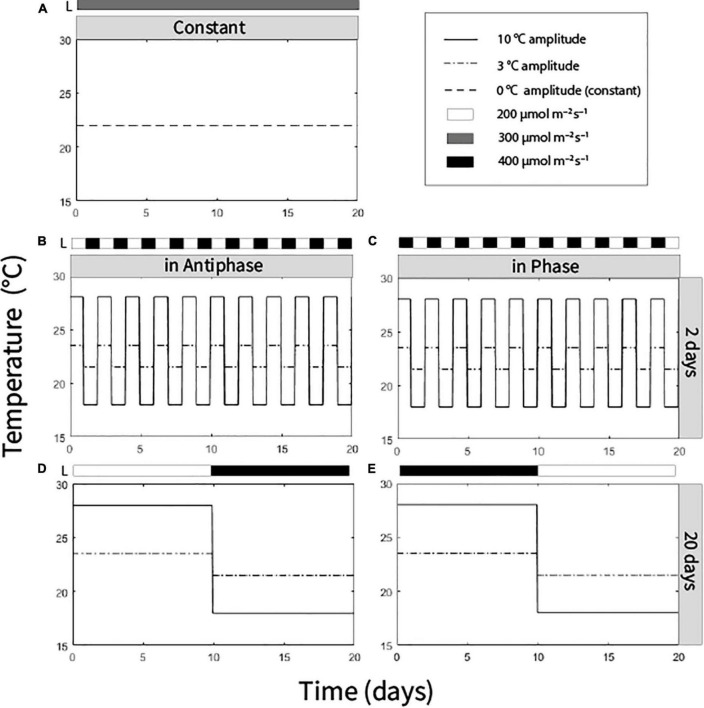
Temperature and light regimes applied in nine treatments: **(A)** Average temperature 22°C (0°C temperature amplitude) and light intensity (300 μmol m**^–^**^2^ s**^–^**^1^) were constantly maintained. **(B)** Antiphase treatments (low light with high temperature followed by high light with low temperature) in a 2-day integration period at two temperature amplitudes (3 or 10°C). **(C)** Phase treatments (high light with high temperature followed by low light with low temperature) in a 2-day integration period at two at two temperature amplitudes (3 or 10°C). **(D)** Antiphase treatments in a 20-day integration period at two temperature amplitudes (3 or 10°C). **(E)** Phase treatment in a 20-day integration period at two temperature amplitudes (3 or 10°C). All treatments received the same light intensity average (300 μmol m**^–^**^2^ s**^–^**^1^) and same temperature average (22°C) at the end of the experiment after 20 days.

We distributed the plants in a multifactorial experiment with three factors with two levels each and a control treatment (hence nine treatments). The factor temperature amplitude (TA) had two levels: (1) 10°C TA (day/night temperatures of 28/25°C or 18/15°C) and (2) 3°C (24.5/21.5°C or 21.5/18.5°C). Integration period (IP), meaning the period of time over which temperature and light was averaged, had two levels: 20 days (light and temperature levels were switched after 10 days) and 2 days (light and temperature levels were switched every day). Temperature was adjusted to light in two ways: in Phase, high temperature at high light intensity, low temperature at low light intensity; or Antiphase: low temperature at high light intensity, high temperature at low light intensity. High light intensity was 400 μmol m^–2^ s^–1^ and low light intensity was 200 μmol m^–2^ s^–1^ (at the top of the canopy) (Figures 1B–E). A ninth treatment with an average daily temperature of 22°C (23/20°C day/night) and constant light intensity of 300 μmol m^–2^ s^–1^ was also performed (Figure 1A). All treatments had received the same average light intensity (300 μmol m^–2^ s^–1^) and the same average temperature (22°C) at the end of the experiment (day 20). In all treatments air humidity was 70%, photoperiod was 16 h and no CO_2_ enrichment was applied (Supplementary Figures 1–3).

### Destructive measurements

For each cabinet, six plants were destructively measured to determine their leaf and stem dry mass (ventilated oven, 72 h at 80°C) and leaf area (LI-3100 area meter, Li-Cor) at days 0, 5, 10, 15, and 20. Because we wanted to investigate responses with a closed canopy, density was changed every 5 days: 59 plants m^–2^ from day 1 to 5, 50 plants m^–2^ from day 5 to 10, 38 plants m^–2^ from day 10 to day 15, and 26 plants m^–2^ from day 15 to day 20.

#### Starch and soluble sugar content

Leaf samples for total soluble sugars and starch content were taken on day 0 (before treatment started) and on days 5, 10, 15, and 20. Leaf samples were taken at the end of the light period. Sampling was done on every other leaf from the bottom to the top to obtain a “canopy” sample. In each selected leaf, one leaflet adjacent to the terminal leaflet was collected. For every treatment (which was repeated two times) six replicate plants per cabinet were taken at each time point (therefore, each time point consisted of 18 samples). Samples were placed in vials, flash frozen in liquid nitrogen and stored at −80°C for further analysis.

Approximately 15 mg of ground leaf material was mixed with 5 ml 80% EtOH (ethanol) in a shaking water bath at 80°C for 20 min for the sugar extraction. After centrifugation at 8500 × *g* for 5 min, 1 ml of the supernatant containing soluble sugars was vacuum dried using a Savant SpeedVac rotary evaporator (SPD2010; Thermo Fisher Scientific, Waltham, MA, United States) and dissolved in 1 ml Mili-Q water and diluted 20× for analysis of soluble sugars. Sucrose, fructose, and glucose quantification was done using a high-performance ion chromatograph (ICS-5000; Thermo Fisher Scientific) with an anion CarboPac 2 × 250 mm exchange column (PA1; Thermo Fisher Scientific) at 25°C with 100 nM NaOH as eluent at the flow rate of 0.25 ml min^–1^. Pulsed amperometry was used for detection and Chromeleon (Thermo Fisher Scientific) was used for analysis of the chromatograms and quantification of sugar concentrations. The remaining pellet after sugar extraction was used for starch determination. After discarding the supernatant that contained the soluble sugars, the remaining pellet was washed three times with 80% ethanol, each time followed by 5 min centrifugation and removal of the supernatant. The remaining pellet was dried for 20 min in a SpeedVac rotary evaporator and resuspended in 2 ml 1 mg ml^–1^ thermostable α-amylase solution (SERVA Electrophoresis, Heidelberg, Germany) and incubated for 30 min at 90°C. Then, 1 ml of 0.5 mg ml^–1^ amyloglucosidase (10115; Sigma-Aldrich) in 50 mM citrate buffer (pH 4.6) was added and the mixture incubated for 15 min at 60°C so that the starch in the sample was converted into glucose. After centrifugation for 5 min at 8500 × *g*, 1 ml of the supernatant was diluted 50× and was used for quantification of glucose content as described above. Glucose levels were analyzed with the HPIC, which this time was eluted with is 100 mM NaOH^+^ 25 mM sodium acetate.

### Statistical analysis

The experiment was carried out in a complete randomized block design with nine treatments. The complete experiment was conducted three times (three blocks). Each time the experiment was conducted; all treatments were randomized over four climate cabinets (experimental unit). The border plants were not used for the study. All data was analyzed in R 4.0.2. Subsequently, significance of the main effects and interactions at each time point was tested using a three-way ANOVA model for the complete factorial design so excluding the control treatment (constant conditions). The statistical tests were all conducted at a probability level of α = 0.05 applying Fishers protected LSD test for mean separation. Differences between treatment means and constant conditions were tested using the LSD.

## Results

### Effect of light and temperature fluctuations on plant growth and morphology

The effect of adapting light to temperature in Phase (high light with high temperature followed by low light with low temperature) or in Antiphase (low light with high temperature followed by high light with low temperature) on total dry weight depended on the integration period (*P* = 0.05, Table 1). For treatments with an integration period of 20 days, Antiphase resulted in 12% higher plant total dry weight (although not statistically significant), 35% higher stem dry weight and 28% longer stem, 4% lower leaf mass fraction compared to Phase (Table 1). For treatments with an integration period of 2 days, Antiphase resulted in 12% lower plant total dry weight, 20% lower stem dry weight, and 15% shorter stem compared to Antiphase (Table 1). Constant conditions had 11–12% less plant total dry weight, 26% less stem dry weight, and 20–22% shorter stem compared to Antiphase 20 days and Phase 2 days, respectively. Total plant dry weight, stem dry weight, stem length, and leaf mass fraction were not statistically significantly different between Constant and Antiphase 2 days and Phase 20 days (Table 1, Supplementary Table 1). SLA and structural dry mass were not statistically different between treatments (Table 1).

**TABLE 1:** Effect of adapting light to temperature in Phase (high light with high temperature followed by low light with low temperature) or in Antiphase (low light with high temperature followed by high light with low temperature) and Integration Period (either 2 or 20 days), averaged over 2 temperature amplitudes (3 or 10°C) on total dry weight, stem dry weight, structural dry weight, NSC, leaf dry weight, stem dry weight, stem length, LMF and SLA of tomato plants at day 20.

In Phase/Antiphase	Integration period (days)	Total dry weight (g DM plant ^–1^)	Structural dry weight (g SDM plant ^–1^)	NSC (gCH_2_O plant^–1^)	Leaf dry weight (g plant^–1^)	Stem dry weight (g plant^–1^)	Stem length (cm)	Leaf mass fraction (LMF)	Specific leaf area (SLA) (cm ^2^ g^–1^)
Phase	2	8.31 a	7.62	0.859 b	6.69	1.62 b	37.3 bc	0.805 ab	286
Antiphase	2	7.32 a	6.33	0.987 b	6.03	1.29 a	32.0 ab	0.825 bc	278
Phase	20	7.31 a	6.80	0.567 a	6.53	1.24 a	31.1 a	0.836 c	295
Antiphase	20	8.19 a	6.66	1.54 c	6.53	1.65 b	38.8 c	0.798 a	259
Constant	Constant	7.31 a	6.44	0.873 b	6.09	1.22 a	30.2 a	0.833 bc	269
F-probability interaction (Phase × Period)		0.050	0.201	0.011	0.185	<0.001	0.005	<0.001	0.321
Standard error of the mean (SEM)		0.626	0.611	0.194	0.558	0.114	2.78	0.009	18.80
LSD (*P* = 0.05)		1.36		0.2053		0.22	5.9	0.009	

In a fifth treatment (Constant) average daily temperature and light intensity were maintained constantly. Data are means of three blocks with six replicate plants per block and averaged over 2 temperature amplitudes (so each value based on 36 plants). Different letters indicate significant differences between treatments; Fisher’s LSD test, (*P* = 0.05).

Plants grown in Phase had 11% higher leaf area compared to Antiphase (Table 2). Constant conditions had 13% less leaf area compared to Phase, but there was no difference with Antiphase (Table 2). Plants grown at lower temperature amplitudes (3°C) had 21% higher leaf area and 18% higher specific leaf area compared to plants grown at 10°C amplitude (Table 3). Constant conditions had 17% less leaf area and specific leaf area compared to 3°C amplitude but did not differ from Antiphase (Table 3).

**TABLE 2:** Effect of adapting light to temperature in Phase (high light with high temperature followed by low light with low temperature) or in Antiphase (low light with high temperature followed by high light with low temperature) on leaf area of young tomato plants at day 20.

In Phase/Antiphase	Leaf area (cm^2^)
Phase	1,822 a
Antiphase	1,635 b
Constant	1,593 b
F-probability main effect (Phase/Antiphase)	0.032
Standard error of the means (SEM)	80.4
LSD (*P* = 0.05)	168

In a third treatment (Constant) average daily temperature and light intensity were constantly maintained. Data are means of 3 blocks with 6 replicate plants per block and averaged over two temperature amplitudes and 2 integration periods (so each value is based on 72 plants). Different letters indicate significant differences between treatments; Fisher’s LSD test, (*P* = 0.05).

**TABLE 3:** Effect of temperature amplitude (3 or 10°C) on leaf area and specific leaf area of young tomato plants at day 20.

Temperature amplitude (°C)	Leaf area (cm^2^)	Specific leaf area (cm^2^ g^–1^)
3	1,899 a	303 a
10	1,558 b	256 b
Constant	1,593 b	269 b
F-probability main effect (Amplitude)	<0.001	0.005
Standard error of the means (SEM)	80.4	13.86
LSD (*P* = 0.05)	142	28

In a third treatment (Constant) average daily temperature and light intensity were constantly maintained. Data are means of three blocks (*n* = 3) with six replicate plants per block and averaged over Phase and Antiphase and two integration periods (so each value is based on 72 plants). Different letters indicate significant differences between treatments; Fisher’s LSD test, (*P* = 0.05).

For treatments with an integration period of 20 days, during the period from days 10 to 15, Antiphase had a 58 and 46% higher growth rate compared to Phase and Constant conditions respectively, (Table 4). For treatments with an integration period of 2 days during the same time interval, Phase had 70 and 25% higher growth rate compared to Antiphase and Constant conditions, respectively (Table 4). Constant conditions had a relatively constant growth rate at all-time intervals (Table 4, Supplementary Figure 4).

**TABLE 4:** Effect of adapting light to temperature in Phase (high light with high temperature followed by low light with low temperature) or in Antiphase (low light with high temperature followed by high light with low temperature) and integration period (either 2 or 20 days), averaged over 2 temperature amplitudes (3 or 10°C) on growth rate of tomato plants over time.

In Phase/Antiphase	Integration period	Growth rate (gDM m^–2^d^–1^) Day 0 to 5	Growth rate (gDM m^–2^d^–1^) Day 5 to 10	Growth rate (gDM m^–2^d^–1^) Day 10 to 15	Growth rate (gDM m^–2^d^–1^) Day 15 to 20
Phase	2	12.8	13.9	17.3 bc	13.2
		(*320/22.65*)	(*280/21.35*)	(*320/22.65*)	(*280/21.35*)
Antiphase	2	9.96	14.1	10.0 a	13.4
		(*280/22.65*)	(*320/21.35*)	(*280/22.65*)	(*320/21.35*)
Phase	20	14.8	13.6	12.7 ab	10.8
		(*400/25.3*)	(*400/25.3*)	(*200/18.8*)	(*200/18.8*)
Antiphase	20	9.15	13.9	20.1 c	11.8
		(*200/25.3*)	(*200/25.3*)	(*400/18.8*)	(*400/18.8*)
Constant	Constant	9.20	14.4	13.8	11.6
		(*300/22*)	(*300/22*)	(*300/22*)	(*300/22*)
F-probability interaction (Phase × Period)		0.380	0.857	0.004	0.873
Standard error of the means (SEM)		2.21	1.68	3.01	3.36
LSD (*P* = 0.05)				6.46	

In a fifth treatment (Constant) average daily temperature and light intensity were maintained constantly. Data are means of three blocks with six replicate plants per block and averaged over 2 temperature amplitudes (so each value based on 36 plants). Light intensity (μmol m^–2^ s^–1^) and temperature (°C) are indicated in brackets. Different letters indicate significant differences between treatments; Fisher’s LSD test, (*P* = 0.05).

### Non-structural carbohydrate dynamics

**FIGURE 2:**
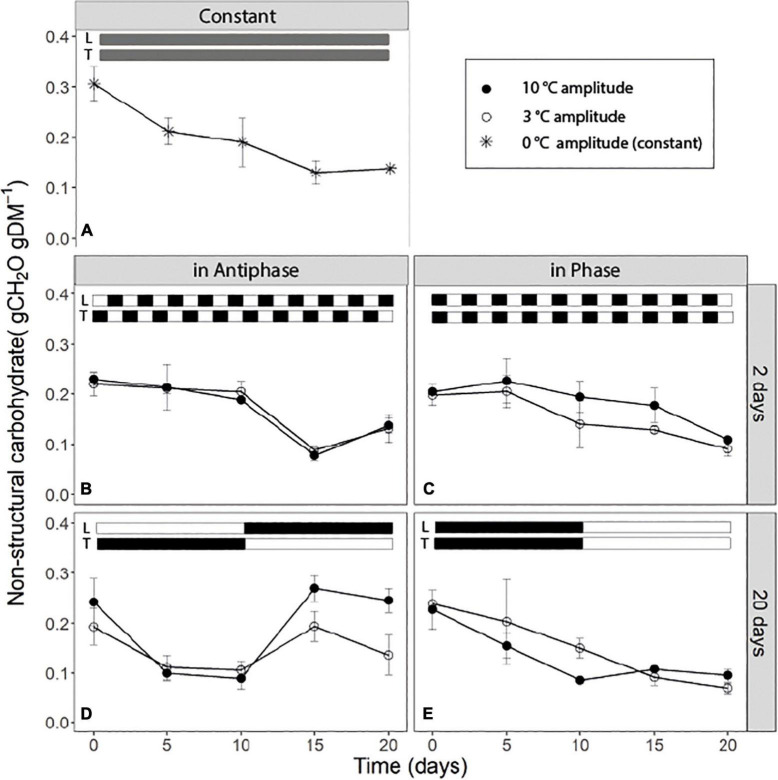
Soluble sugar content of tomato leaves (sum of glucose, fructose, and sucrose) over time for **(A)** constant light (300 μmol m**^–^**^2^s**^–^**^1^) and temperature (22°C), **(B)** light and temperature in Antiphase and an integration period of 2 days (200 μmol m**^–^**^2^s**^–^**^1^ and 28°C for 1 day followed by 400 μmol m**^–^**^2^s**^–^**^1^ and 18°C for 1 day), **(C)** light and temperature in Phase and an integration period of 2 days (400 μmol m**^–^**^2^s**^–^**^1^ and 28°C for 1 days followed by 200 μmol m**^–^**^2^s**^–^**^1^ and 18°C for 1 day), **(D)** light and temperature in Antiphase and an integration period of 20 days (200 μmol m**^–^**^2^s**^–^**^1^ and 28°C for 10 days followed by 10 days at 400 μmol m**^–^**^2^s**^–^**^1^ and 18°C), and **(E)** light and temperature in Phase and an integration period of 20 days (400 μmol m**^–^**^2^s**^–^**^1^ and 28°C for 10 days followed by 10 days at 200 μmol m**^–^**^2^s**^–^**^1^ and 18°C). Closed symbols are treatments with a temperature amplitude of 10°C and open symbols a temperature amplitude of 3°C. White bars above the graphs indicate a low level of light intensity and temperature and black bars indicate a high level of light intensity and temperature. Data are means of three blocks (*n* = 3) with 6 replicate plants per block. Error bars are ± SEM.

For treatments with an integration period of 20 days, Antiphase (low light with high temperature followed by high light with low temperature) showed a steep decline in NSC from about 0.25 g CH_2_O g^–1^DM on day 0 to about 0.10 g CH_2_O g^–1^ DM on day 5 at both temperature amplitudes (3 and 10°C). The NSC concentration remained constant from days 5 to 10 (Figure 2D). From day 10 onward, when light intensity increased and temperature decreased, NSC increased again to 0.25 g CH_2_O g^–1^ DM for the 10°C amplitude and to 0.14 g CH_2_O g^–1^DM for 3°C amplitude (Figure 2D). Treatments in Phase (high light with high temperature followed by low light with low temperature) showed a linear decrease in the NSC from day 0 to 10 in both temperature amplitudes (3 and 10°C). When temperature amplitude was large (10°C), the minimum NSC concentration (0.1 g CH_2_O g^–1^DM) was reached at day 10, and afterward the concentration remained constant. The NSC decreased at a slower rate from day 0 to 10 in small temperature amplitudes (3°C) and the minimum (0.1 g CH_2_O g^–1^DM) was reached on day 15 (Figure 2E). For treatments with an integration period of 2 days, Antiphase maintained almost a constant NSC concentration from day 0 to 10. After day 10, the concentration decreased on days with high temperature and low light intensity and increased again at day 20 on a day with low temperature and high light intensity for both temperature amplitudes (Figure 2B). Treatments in Phase showed a general trend of a decline in the NSC pool (from 0.20 to 0.1 gCH_2_O gDM^–1^) throughout the experiment (Figure 2). Plants grown under constant conditions showed a continuous decrease in NSC until day 15 (reaching a minimum of 0.14 gCH_2_O gDM^–1^) and afterward, the NSC concentration remained constant (Figure 2).

### Soluble sugar dynamics

**FIGURE 3:**
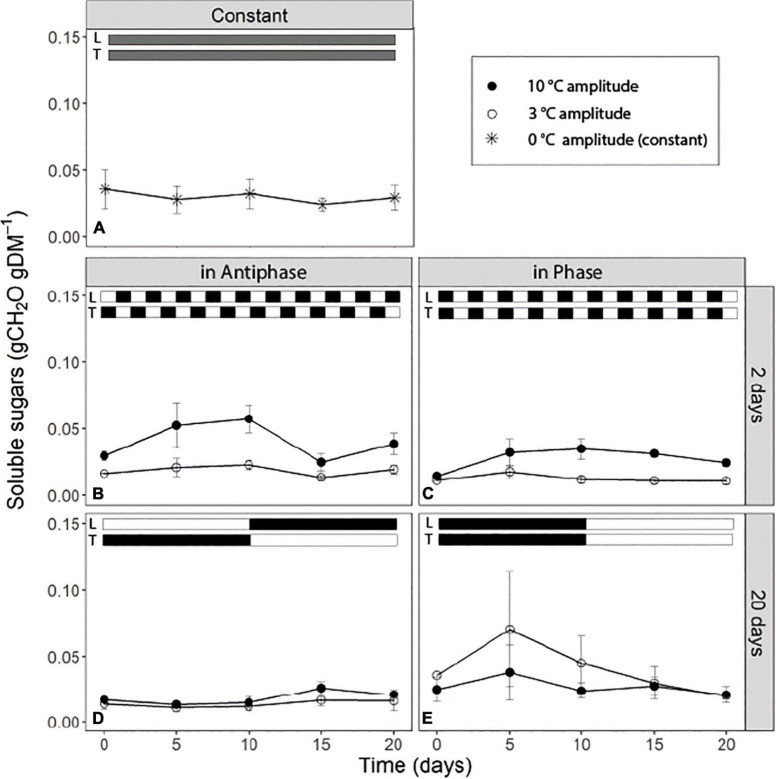
Time course of non-structural carbohydrate content of tomato leaves (sum of glucose, fructose, sucrose and starch) for **(A)** constant light (300 μmol m**^–^**^2^s**^–^**^1^) and temperature (22°C), **(B)** light and temperature in Antiphase and an integration period of 2 days (200 μmol m**^–^**^2^s**^–^**^1^ and 28°C for 1 day followed by 400 μmol m**^–^**^2^s**^–^**^1^ and 18°C for 1 day), **(C)** light and temperature in Phase and an integration period of 2 days (400 μmol m**^–^**^2^s**^–^**^1^ and 28°C for 1 days followed by 200 μmol m**^–^**^2^s**^–^**^1^ and 18°C for 1 day), **(D)** light and temperature in Antiphase and an integration period of 20 days (200 μmol m**^–^**^2^s**^–^**^1^ and 28°C for 10 days followed by 10 days at 400 μmol m**^–^**^2^s**^–^**^1^ and 18°C), and **(E)** light and temperature in Phase and an integration period of 20 days (400 μmol m**^–^**^2^s**^–^**^1^ and 28°C for 10 days followed by 10 days at 200 μmol m**^–^**^2^s**^–^**^1^ and 18°C). Closed symbols are treatments with a temperature amplitude of 10°C and open symbols a temperature amplitude of 3°C. White bars above the graphs indicate a low level of light intensity (L) and temperature (T) and black bars indicate a high level of light intensity (L) and temperature (T). Data are means of 3 blocks (*n* = 3) with six replicate plants per block. Error bars are ± SEM.

For treatments with an integration period of 20 days Antiphase, at both temperature amplitudes, had on average lower SS concentration (0.015 g CH_2_O g^–1^DM) compared to Phase (Figures 3D,E). Antiphase treatment at a 20-day integration period showed a slight increase in the SS sugar at day 10 at 10°C temperature amplitude once PPFD increased from 200 to 400 μmol m^–2^s^–1^ and temperature decreased from 28 to 18°C (Figure 3D). For treatments with an integration period of 2 days, soluble sugars were on average twice as high at 10°C amplitude, compared with 3°C amplitude no matter whether in Phase of Antiphase (Figures 3B,C). Constant conditions resulted in constant SS concentration over time (0.03 g CH_2_O g^–1^DM) (Figure 3A). In all treatments, SS rarely were above 0.06 gCH_2_O gDM^–1^.

## Discussion

### Carbon storage and remobilization largely buffer the effects of temperature and light fluctuations on growth

Carbon reserves are hypothesized to play a fundamental role in a plant’s coping with environmental fluctuations, at different temporal scales from within a day to seasons (Legros et al., 2009; Dietze et al., 2014). Short-term temperature and light fluctuations (every other day) lead to a similar time course (both a depletion/decrease) of the NSC over 20 days independent from whether temperature and light intensity are in Phase or in Antiphase (Figures 2B,C). Constant conditions also lead to a depletion of the NSC over time, although in this case, the concentration of NSC start declining at a rate of ∼0.012 gNSC day^–1^ from day 0 to 5, compared to Phase or Antiphase where during this time period, there is almost no depletion. This may indicate that plants in constant conditions were source-limited already from day 0 and had no C to allocate to storage. Surprisingly, differences in structural dry weight between these treatments were relatively small considering such contrasting temperature and light fluctuations (Table 1). Still, on the short-term, the dynamics of the NSC pools followed the patterns of light and temperature: plants reduced the amount of stored NSC on days with a low supply relative to the demand (i.e., days with low light intensity and high temperature) for example at day 15 (Figure 2B) and accumulated NSC carbohydrates over days with an excess supply (i.e., days with a high light intensity and a low temperature) for example at day 20 (Figure 2B). While on the short term we can see patterns of accumulation and depletion, on the long term we can see a continuous loss in the NSC pools indicating a net depletion of the storage pools, meaning that plants were source limited. If plants were grown at higher light intensities where source is lower than the sink, then gradual increases on NSC are expected up to the moment when the NSC pool reaches its limit, or until plants become source-limited again and NSC pool starts depleting.

### Plants build up non-structural carbohydrates reserves when supply exceeds demand up to a maximum

Fluctuations in the NSC pool are mainly driven by changes in assimilation vs. growth and respiration, which in turn are highly dependent on temperature and light environment. We hypothesize that a low light intensity results in reduced assimilation, and when this coincides with high growth and respiration demands due to high temperature, then C storage is quickly depleted. Conversely, a situation with increased assimilation rate due to high light intensity and low growth or respiration demand due to reduced temperature will result in a build-up of NSC storage. Our results show that NSC storage pools were able to recover after 5 days (i.e., Figure 2D from day 10 to 15), (accumulation of three times more compared to the minimum NSC observed) from the C-limiting conditions after an increase in the light intensity. The rate of accumulation depended on temperature, for example, at 18°C the accumulation rate was 0.038 gNSC day^–1^, while at 21.5°C the accumulation rate was lower with 0.014 gNSC day^–1^ (Figure 2D from day 10 to 15). Similarly, Klopotek and Kläring (2014) showed that once plants were moved from 26 to 16°C (at 400 μmol m^–2^s^–1^), in only 2 days, plants were able accumulate three times as much starch compared to the concentrations measured just before the temperature change. A similar behavior has been long observed for *Arabidopsis* on a diurnal time scale (Smith and Stitt, 2007; Gibon et al., 2009; Sulpice et al., 2014; Mengin et al., 2017) for example, when during short days plant accumulate NSC (mainly in the form of starch) and these reserves are used during the C limiting conditions of “darkness” (the night). The question remains: what are the limits for C storage in which growth then becomes negatively affected? Most likely conditions in the present experiment were not extreme enough compared to starvation conditions, which simply did not trigger or impair growth of the plants. In a more extreme case, Weber et al. (2018) showed that tissue concentrations of C reserves decreased in complete darkness, but seedlings were able to recover quickly after a few days of re-illumination. However, in this case, after re-illumination, the rebuilding of C reserves of seedlings was prioritized over other C-sink activities such as growth (Weber et al., 2018, see **Figures 5, 6**).

An increased growth rate was observed at long integration periods (20 days), immediately after the increase in light intensity (Table 4) and coincides with a period of high accumulation of NSC (Figure 2D). This suggests that the stored NSC is used to give a higher growth rate for a few days. This was previously observed by Gent (1986) in tomato plants, where RGR was 43% higher and NSC content was 41.5% higher at plants grown at high light intensity compared to low light intensity. However, in our experiment, after 5 days with a high NSC concentration, the NSC pool and growth rates started declining, indicating a possible feedback inhibition of photosynthesis, and suggesting that the NSC pool reached a ‘limit’ at ∼0.28 gCH_2_O gDM^–1^. Altogether, this shows that NSC pools respond in a time scale of days to C source-sink asynchronies and that C storage and remobilization largely buffer the effects of temperature and light fluctuations on plant structural growth.

### Stored non-structural carbohydrates were not entirely remobilized even under source-limited conditions

Previous studies have shown that plants keep a relatively high minimum NSC concentration at all times (30 to 50% the percentage from the seasonal maximum) (Martínez-Vilalta et al., 2016) unless they are under extreme conditions leading to death (Weber et al., 2018). From our results we can see that in all treatments, the NSC pool seems to reach a minimum, roughly 33% from the maximum NSC observed during the experiment which goes in line with previous research across different species and climates (Martínez-Vilalta et al., 2016). In our study, if light intensity and temperature remain the same, this “minimum” level is maintained (Figure 2). This simply reflects that at this point, plants are not allocating any new C to storage; however, they are also not using any reserve although still 10% of the total plant mass is NSC (Figure 2). From the remaining NSC, only around 30% are soluble sugars (Figure 3) which denotes that there is still a fraction of starch that remains stored despite that the C flows for respiration and growth could be potentially higher than C assimilation (for example Figure 2D, day 5 to 10). Hence there is a fraction of the NSC that is non-mobilizable, and that plants are likely keeping this level to prevent acute depletions of the NSC at all times, unless conditions are extreme.

### Short-term fluctuations increase the concentration of soluble sugars when temperature fluctuations are large

Unexpectedly, short-term (days) fluctuations of light and temperature lead to constantly higher concentrations of soluble sugars in the leaves when temperature fluctuations were large (Figures 3B,C) (i.e., when mean daily temperature fluctuated between 18 and 28°C) compared to a mean daily temperature fluctuating between 21.5 and 23.5°C. Soluble sugars are involved in the response to a number of stresses, as they act as signaling molecules (Couée et al., 2006). Under low temperatures, accumulation of soluble sugars is typical as they contribute to the stabilization of the osmotic cell potential (Martínez-Vilalta et al., 2016; Pommerrenig et al., 2018) and they have a protective role against ROS (Keunen et al., 2013). Perhaps those days with low temperatures triggered a “cold acclimation” type of response in plants, or reduced respiration contributed to the accumulation of soluble sugars. However, in that case we would also observe such a response in treatments where plants were exposed to 18°C constantly for 10 days (Figures 3D,E), but we did not observe this. Most likely temperatures were simply not sufficiently low to trigger a cold acclimation or to reduce respiration substantially. Another explanation is that plants perceived the “repetitive sudden changes” in temperature as a sign of stress and triggered a response that led to an accumulation of soluble sugars. We conclude that daily abrupt changes in temperature lead to an accumulation of soluble sugars. Although extensive research has been conducted regarding accumulation of sugars as a response to low temperature (for a review see Ruelland et al., 2009) or excess light (Schmitz et al., 2014) there is limited research on the response of the soluble sugar pool to dynamic fluctuations in light and temperature and its interaction which is relevant for plants exposed to naturally occurring environmental fluctuations.

Our findings also support the dual role of NS in plants: starch fluctuates and acts as a storage for future use under C-limiting conditions or drought, while soluble sugars stay relatively constant to perform immediate metabolic functions and are kept above some critical threshold (Sala et al., 2012; Dietze et al., 2014; Martínez-Vilalta et al., 2016).

### Could temperature dependence of CO_2_ assimilation explain higher growth in Phase treatments at short-term fluctuations?

The effect of adapting light to temperature in Phase or in Antiphase on total dry weight depends on the integration period (Table 1). When temperature and light changed every other day, adapting light to temperature in phase led to 14% higher total dry weight, compared to adapting light to temperature in Antiphase. These results could not be explained by changes in leaf morphology or allocation, as SLA, LA or LMF did not significantly differ between treatments (Table 1). As discussed earlier, the rate of depletion of the NSC was almost identical, so accumulation and remobilization of NSC could not explain the differences either. It is possible that the influence of temperature on CO_2_ assimilation plays a significant role, as in many species, the optimal temperature that maximizes leaf photosynthetic rate increases with increasing growth temperature (Hikosaka et al., 2006). Most likely, carbon assimilation was temperature limited for Antiphase treatments, when plants were grown at days with high light intensity (400 μmol m^2^ s^–1^) and low temperature (18°C), where according to Thornley (2016, Figure 2), temperature was below optimum for the given light intensity. The next day, when plants were exposed to a low PPFD (200 μmol m^2^ s^–1^) but a relatively high temperature (28°C) carbon assimilation was simply reduced because of the lower PPFD but the higher temperatures lead to an increased respiration. If this pattern is then repeated over time (e.g., 20 days) this may have led to a disadvantage compared to fluctuations where the PPFD coincides paired with the optimal temperature for photosynthesis, which is the case in the Phase treatment.

A similar reasoning could be followed for the temperature dependence of CO_2_ assimilation at different CO_2_ levels, where there is a shift to a higher optimum temperature at elevated CO_2_ levels (Körner et al., 2009). While in our study we maintained CO_2_ at ambient concentrations, in a greenhouse where supplementing with CO_2_ is a common practice, these interactions must be considered.

### In long integration periods, ending with a high light intensity leads to a large total dry weight

When temperature and light changed every 10 days, adapting light to temperature in Antiphase led to 12% higher total dry weight, compared to adapting light to temperature in phase. When plants grown at low light intensities (200 μmol m^–2^ s^–1^) were switched to a high light intensity (400 μmol m^–2^ s^–2^), there is a 44% increased growth rate, compared with plants grown first at high light intensities and then switched to low light intensities (Table 4). This was expected, as an increase in the light intensity (and therefore in the photosynthesis rate) leads to an increase in the growth rate (Walters, 2005), however, the increased growth rate was only sustained for 5 days, and thereafter the growth rate was reduced from 20.1 to 11.8 gDM m^–2^. The reduction in growth rate in days 15–20 was also accompanied by a reduction in the NSC pool (Figure 2D). A possible explanation is that initially, the increase in light intensity led to an increased growth rate, but after a while the NSC buffer was “completely full” resulting in feedback inhibition of photosynthesis, which led to both, a decrease in the NSC reserves and also in the growth rate (Paul and Foyer, 2001; Gent and Seginer, 2012).

In both treatments (Phase and Antiphase), temperature changed equally (Figures 1E,D) (high temperature during the first 10 days and low temperature during the last 10 days), therefore we can exclude temperature as a factor explaining the increased growth rate. A remaining question is: would the growth rate be equally higher if the switch to higher light intensities would be paired with an increased temperature? Interestingly, in an additional experiment with an integration period of 14 days we observed similar increase in growth rate (2.2 and 2.8 times higher) for the treatment that ended high light intensity (400 μmol m^–2^ s^–2^) and low temperature (18°C) and treatment that ended with high light intensity but also high temperature (28°C) (Supplementary Table 2, Supplementary Figure 5). The main differences were that at low temperatures, around 42% of the total dry weight was allocated to storage (starch and soluble sugars) while at high temperatures only 25% is allocated to storage (Supplementary Table 3).

Accumulation of structural dry mass was not different between Antiphase and Phase at day 20 (differences less than 0.12 gSDM plant^–1^), However, the absolute size of the non-structural carbohydrate pool is three times larger, compared to Phase (Table 1). The implications of these results are that the greater the mass of reserves, the higher the C availability is to build new tissue (Wiley et al., 2019). In this case, if these plants would then be exposed to an environmental stress such as high temperatures, drought, or high salinity, they would have more resources available to remobilize and release energy and sugars to help mitigate the stress (Thalmann and Santelia, 2017).

### Fluctuating temperature led to higher leaf area and specific leaf area

In our study we observed changes in leaf morphology upon fluctuating temperature. Fluctuating temperature with an amplitude of 3°C reduced leaf thickness and increased leaf area (Table 3) compared with larger temperature fluctuations (10°C) or constant conditions. A similar response was observed in plants grown under fluctuating light regimes (Vialet-chabrand et al., 2017; Zhang and Kaiser, 2020). Additionally, when plants are grown under fluctuating light and temperature, adjusting light to temperature in Phase (for example, high light levels together with high temperature levels) results in a larger leaf area compared to adjusting light to temperature in Antiphase (high light levels together with low temperature levels) or with constant conditions (Table 2). This is a remarkable response, as an increase in leaf area or specific leaf area can lead to a larger light capture per unit biomass, ultimately leading to a higher dry weight accumulation. This implies that constant conditions (similar regimes used in greenhouse production or climate chambers) are not necessarily optimal, at least for tomatoes.

### Future implications

Whereas much of the earlier research focused on seasonal patterns of C accumulation and remobilization (Sala et al., 2012; Tixier et al., 2013; Huang et al., 2018; Weber et al., 2019) or diurnal C remobilization (starch degradation pattern) (Gibon et al., 2004; Graf et al., 2010; Scialdone et al., 2013; Pilkington et al., 2015), future studies should explore in more detail the day to day effect of fluctuations on the C pool dynamics and how these relate to growth. For example: what are the rates of accumulation and depletion of C and to what extent do these depend on temperature? Or where are the thresholds of minimum and maximum NSC before we can observe detrimental effects on growth or prioritization of C accumulation over growth? Future research should include gas exchange measurements, metabolic flux analysis, isotopic techniques and dynamic growth monitoring with the use of sensors (e.g., De Swaef et al., 2013), to have a better integration of the whole plant carbon economy over several days. As there is almost an infinite number of possible combinations of light and temperature fluctuations that lead to different source-sink relationships, parameterizing C accumulation and depletion rates and calibrating a model would allow us to test hypotheses over a larger range of environmental conditions.

Furthermore, we observed that short- and long-term fluctuations in light and temperature influence structural mass accumulation only slightly, and that C storage and remobilization plays a key role in buffering these fluctuations. These results raise the following question: is there a reason to shift our paradigm about the way we grow crops in controlled environments? Throughout the years we have been growing plants in greenhouses and climate rooms highly focused on maintaining the ‘perfect constant’ climate and thus, reducing environmental fluctuations. It has now become increasingly questionable whether this may be the best practice, since plants in nature grow under highly variable conditions at all-time scales (seconds to minutes, diurnally, day to day or seasonally) (Athanasiou et al., 2010; Poorter et al., 2016; Vialet-chabrand et al., 2017). Rapid fluctuations in light (seconds to hours) (e.g., Zhang and Kaiser, 2020; Bhuiyan and Van Iersel, 2021) or daily fluctuations in light and temperature (e.g., Dieleman and Meinen, 2007; Klopotek and Kläring, 2014) may not necessarily have detrimental effects on plant growth and may even improve physiological traits that lead to the same or improved biomass production in plants. Additionally, the fact that crops can buffer fluctuations in light and temperature allow a higher freedom in horticulture for allowing the climate to deviate from the set points. This has a profound impact on the energy use efficiency, as flexible climate set points can reduce energy consumption in greenhouse up to 20% (Körner and Challa, 2004; van Beveren et al., 2015).

## Conclusion

Differences in final structural dry weight were relatively small, while NSC concentrations were highly dynamic and followed changes of light and temperature (a positive correlation with decreasing temperature and increasing light intensity). High temperature and low light intensity lead to depletion of the NSC pool, but NSC level never dropped below 8% of the plant weight and this fraction was not mobilizable. Low temperatures lead to a faster accumulation of NSC and the NSC pool reached a ‘limit’ at ∼0.28 gCH_2_O gDM^–1^. After 5 days with a constantly high NSC concentration, the NSC pool and growth rates started declining, indicating a possible feedback inhibition of photosynthesis. Our results suggest that growing plants under fluctuating conditions do not necessarily have detrimental effects on plant growth and may improve biomass production in plants. These findings highlight the importance in the NSC pool dynamics to buffer fluctuations of light and temperature on plant structural growth.

## Data availability statement

The raw data supporting the conclusions of this article will be made available by the authors, without undue reservation.

## Author contributions

AZ, EH, and LM designed the research. AZ conducted the measurements, drafted the manuscript, and analyzed the data with suggestions from EH and LM. EH and LM made substantial contributions to improve the manuscript. All authors contributed to the article and approved submitted version.
