# Supplementary material for: Non-structural carbohydrate dynamics and growth in tomato plants grown at fluctuating light and temperature
Source: Front Plant Sci. 2022 Oct 3;13:968881. doi: 10.3389/fpls.2022.968881 (PMC9574331; doi:10.3389/fpls.2022.968881)
Supplement: Supplementary file 1 [file Data_Sheet_1.pdf]

Supplementary Material

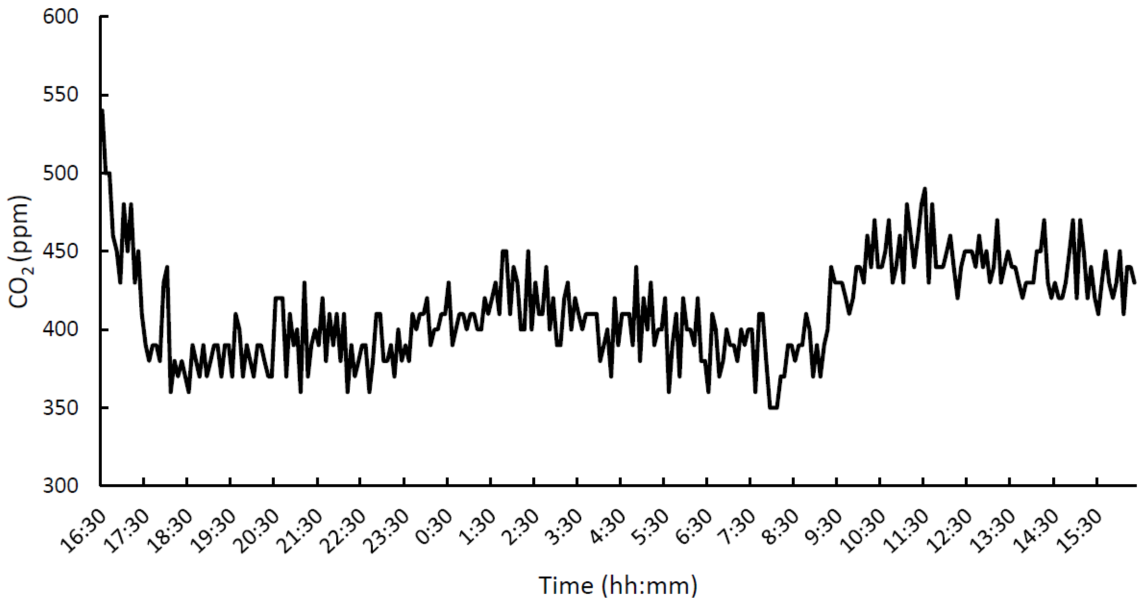

Supplementary Figure 1. Time course of CO2 during a 24-hour measurement on experimental day 12 in growth cabinet 14. Average value over 24 hours is 412 ppm.

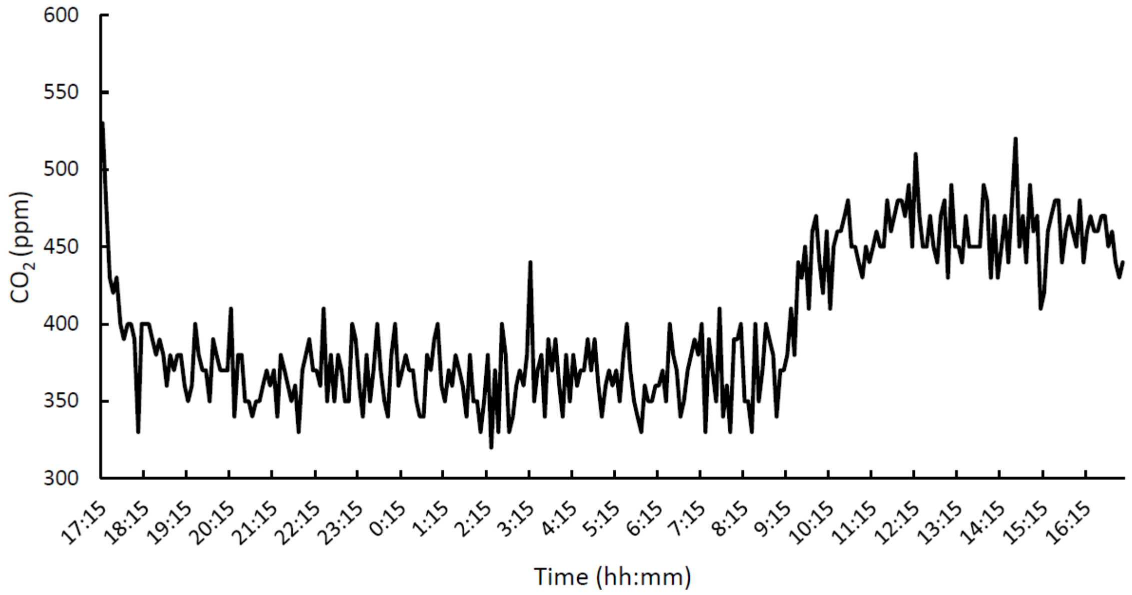

Supplementary Figure 2. Time course of CO2 during a 24-hour measurement on experimental day 13 in growth cabinet 15. Average value over 24 hours is 399 ppm.

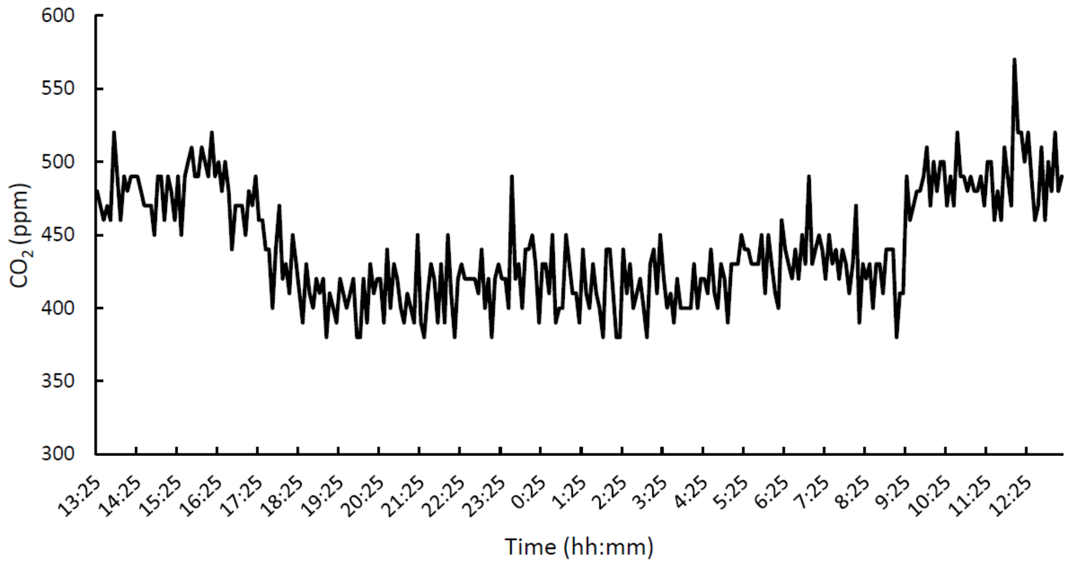

Supplementary Figure 3. Time course of CO2 during a 24-hour measurement on experimental day 12 in growth cabinet 16. Average value over 24 hours is 441ppm

Supplementary Table 1. Effect of adapting light to temperature in Phase (high light with high temperature followed by low light with low temperature) or in Antiphase (low light with high temperature followed by high light with low temperature) and Integration Period (either 2 or 20 days), averaged over 2 temperature amplitudes (3 or 10°C) on total dry weight, stem dry weight, NSC, leaf dry weight and stem dry weight. Data are means of 3 blocks with 6 replicate plants per block and averaged over 2 temperature amplitudes (so each value based on 36 plants).

Phase/Antiphase	Integration Period (days)	Total Dry Weight (g DM m^{-2})	NSC (gCH_2O m^{-1})	Leaf dry weight (g m^{-1})	Stem Dry Weight (g m^{1})
Phase	2	216.1	22.34	173.9	42.12
Antiphase	2	190.3	25.66	156.8	33.54
Phase	20	190.1	14.74	169.8	32.24
Antiphase	20	213.0	40.04	169.8	42.9
Constant	Constant	190.1	22.69	158.34	31.72

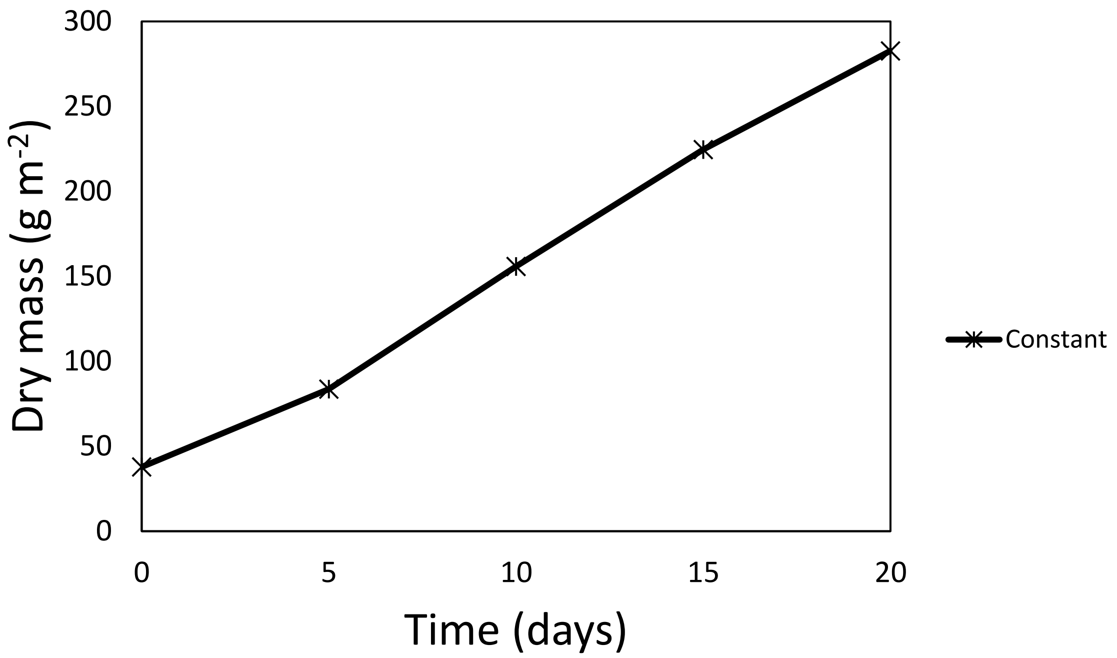

Supplementary Figure 4. Total dry weight over time of tomato plants grown at 300 µmol m^{-2} s^{-1} and 22 °C constantly for 20 days. Symbols indicate the mean of two replications (n=2) and bars are SEM.

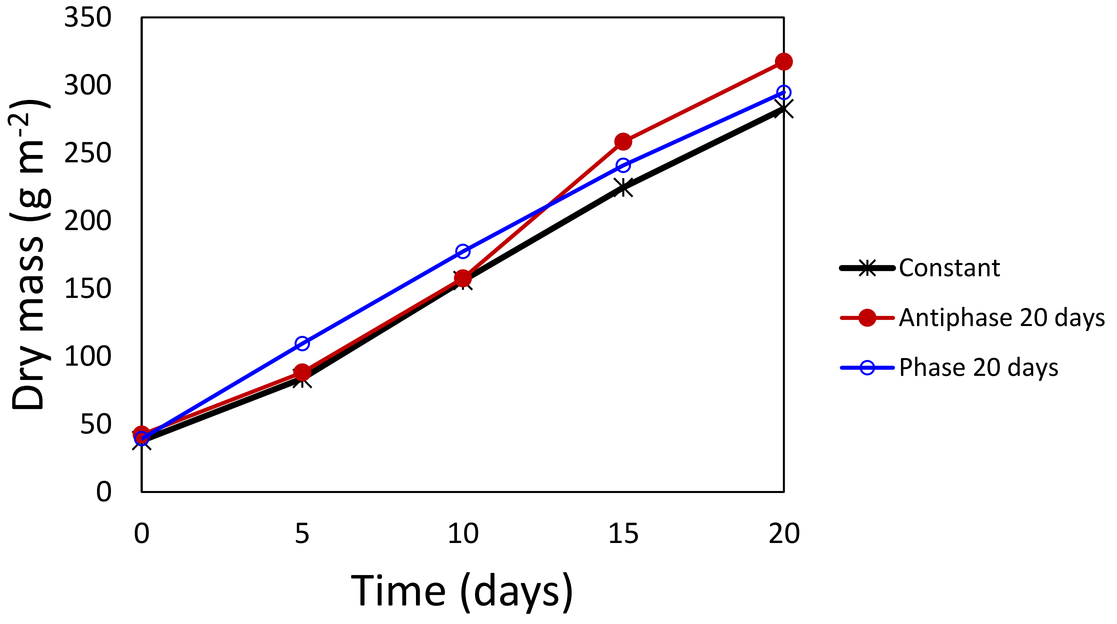

Supplementary Figure 5. Cumulative dry mass of tomato plants over time. Star black symbols stand for constant conditions: constant light (300 µmol $m^{-2}s^{-1}$) and temperature (22 °C), red closed symbols are treatment in Antiphase light and temperature in Antiphase and an integration period of 20 days

(200 µmol $m^{-2}s^{-1}$ and 28 °C for 10 days followed by 10 days at 400 µmol $m^{-2}s^{-1}$ and 18 °C and blue closed symbols is treatments in phase light and temperature in Phase and an integration period of 20 days (400 µmol $m^{-2}s^{-1}$ and 28 °C for 10 days followed by 10 days at 200 µmol $m^{-2}s^{-1}$ and 18 °C. Data are means of 3 blocks (n=3) with 6 replicate plants per block. Error bars are ±SEM.

Supplementary Table 2. Growth rate of tomato plants grown in Phase (400 µmol m^{-2} s^{-1} and 28 °C for 7 days and then at 200 µmol m^{-2} s^{-1} and 18 °C for the last 7 day), Phase low (200 µmol m^{-2} s^{-1} and 18 °C for 7 days and then at 400 µmol m^{-2} s^{-1} and 28 °C for the last 7 day) or in Antiphase (200 µmol m^{-2} s^{-1} and 18 °C for 7 days and then at 400 µmol m^{-2} s^{-1} and 28 °C for the last 7 day).

	Growth rate (gDM d^{-1}) Day 0 to 7	Growth rate (gDM d^{-1}) Day 7 to 14
Phase	9.17	8.91
Antiphase	6.33	14.2
Phase low	5.14	14.9

Supplementary Table 3. Plant dry mass, non-structural carbohydrate and structural dry mass of tomato plants grown in Phase low (200 µmol m^{-2} s^{-1} and 18 °C for 7 days and then at 400 µmol m^{-2} s^{-1} and 28 °C for the last 7 day) or in Antiphase (200 µmol m^{-2} s^{-1} and 18 °C for 7 days and then at 400 µmol m^{-2} s^{-1} and 28 °C for the last 7 day).

	Plant dry mass (gDM m^{-2})		Non-structural carbohydrate (gCH_2O m^{-2})		Structural dry mass (gSDM m^{-2})	
Time	Phase low	Antiphase	Phase low	Antiphase	Phase low	Antiphase
0	40	40	8.28	8.12	31.7	31.9
7	76.0	84.3	16.8	9.61	59.2	74.7
14	180	183	43.5	77.0	136	106
